# Conference Report: ISO/IEC WORKSHOP ON WORLDWIDE RECOGNITION OF OSI TEST RESULTS, Gaithersburg, MD, May 6–8, 1991

**DOI:** 10.6028/jres.096.050

**Published:** 1991

**Authors:** Stephen Nightingale

**Affiliations:** U.S. GOSIP Testing Program, National Institute of Standards and Technology, Gaithersburg, MD 20899

## 1. Reasons for the Workshop

The testing of Open Systems Interconnection (OSI) products is expensive and time consuming. Hence there is a great desire on the part of the suppliers of such products to have their products tested only once. At the same time, clients are seeking assurances that newly acquired OSI products will interwork with their existing systems. These two points of view cannot currently be reconciled in the absence of global arrangements on the recognition of the results of testing OSI products. In order to identify a way to proceed, the Information Technology Resources Support Group (ITRSG) recommended that the International Organization for Standardization (ISO) and the International Electrotechnical Commission (IEC) should organize an International Workshop on the Worldwide Recognition of OSI Test Results.

The Workshop, which was carefully planned by a Program Committee in which most of the interested parties from North America, Europe, and “the rest of the world,” i.e., Australasia, were represented was held at the National Institute of Standards and Technology (NIST) from May 6–8, 1991.

## 2. Summary of the Workshop

The workshop was attended by almost 150 participants and there were 25 speakers coming from a wide range of organizations worldwide, with a variety of stakes in the OSI Test Results issues. On the first day, the objectives were presented by Dr. D. Rayner of the U.K. National Physical Laboratory, and tutorials were given on the state of the art in testing, Laboratory accreditation, and certification, followed by government, industry and commercial user positions on objectives. The second day, which was chaired by Mr. Y. Yokoyama of Japanese INTAP explored current practices in testing, laboratory accreditation, and certification, in the different regions throughout the world. Reports on the European scene, including the European Infrastructure, were presented by the chairmen of ECITC (the European Committee on Information Technology Testing and Certification), OSTC (Open Systems Testing Consortium, An Example of a Recognition Arrangement), and the manager of ACERLI—a French laboratory within the European scheme. The North American scene was represented by talks from NIST, Corporation for Open Systems (COS), and CIGOS-the Canadian Interest Group on Open Systems. The “rest of the world” comprised talks from Japan, Korea, Australia and from the World Federation of MAP/TOP User Groups.

The third day was intended to digest the information from the previous 2 days and provide plans for the future. The workshop was chaired on the third day by Kevin Mills of NIST, who provided a provocative introduction and expanded the scope of the “One Stop World” to encompass: standardization, production and maintenance of standards; harmonization, mutual recognition, validation and equivalence of test systems; accreditation of conformance testing laboratories; certification of conformance tested products; and finally, the role of interoperability testing. Discussions, led by previous speakers, followed a number of strawman proposals.

## 3. Test Methodology Equivalence

Fundamental to the harmonization of test reports is the harmonization of standardized profiles. This work is already underway in the Regional Workshop Coordinating Committee, which mediates the efforts of the Asian and Oceanic Workshop (AOW), the European Workshop for Open Systems (EWOS), and the OSI Implementors’ Workshop (OIW).

A single set of test suites for OSI conformance testing is necessary. In the past, OSI standards have been produced without accompanying means of verifying that implementations can conform. This led to the ad hoc and fragmented production of tests of varying quality, which is the situation as it exists today. The ISO/IEC workshop recognized the need to stimulate and coordinate nonduplicative efforts in filling the gaps left by the formal standardization process. The recent history of funding for conformance testing is that the “public” tests and the test system development efforts have been predominantly European in origin. The workshop noted that funding for test specification development is an issue for each region. The workshop also noted that for future protocol developments, progress should include test suites with, not after, protocol specifications.

In considering the application of quality controls to test system developments, there are different philosophical approaches from different schemes in the world today: the European scheme requires maintaining equivalent test tools and executable test suites to produce equivalent test reports. This would be fine in a world where the test suites are finalized and the test technology is mature. The NIST approach to test system acceptance criteria is more pragmatic—realizing that existing test technology is not mature and test coverage is not 100% of the features of each protocol, we set a baseline for acceptable coverage, and registered those test systems which were above the line. The baseline is increased periodically, to encourage improvements in the testing technology, until the level of full coverage of the protocol features is reached. In this scheme, test report equivalence is reached when a full coverage test suite has been developed, but not in the interim period. The theory behind this approach is that allowing some variation between “acceptable” test systems provides the freedom to stimulate improvements in competing test technologies, which is more important in the short term than maintaining a rigid equivalence of a small sample of the possible tests. The question of harmonizing test system acceptance criteria, to include a reasonable, staged, interim approach was not well explored in the workshop, but the clear message is that this is one of the issues to be handled by the conformance testing special interest group (SIG) of each of the regional implementors’ workshops.

## 4. Mutual Recognition of Accreditation

Perhaps the easiest area to consider harmonizing is that of accreditation, although there was some friction here between the accreditation bodies and the OSI community. The accreditation bodies such as the National Voluntary Laboratory Accreditation Program (NVLAP) in the United States, the National Measurement Accreditation System (NAMAS) in England and Reseau Nationale d’Essais (RNE) in France, and their mediating body the International Laboratory Accreditation Conference (ILAC), have a way of recognizing each others’ programs by bilateral agreements. Traditionally this has been “across the board,” i.e., independent of any particular test method. The network of bilateral agreements in place at the moment is rather sparse. The OSI, and wider Information Technology (IT) communities make the claim that an IT specific interpretation of ISO Guide 25, covering the conduct of laboratory accreditations, is required, and hence wish to influence the accreditation bodies to make sector specific agreements. The principal issue in this case is that the concept of calibration, central to most specimen testing laboratories, is not applicable in IT. Instead, in software testing, the concept of validation of software test systems against a canonical, or a notional model is in force. This “sector specific” movement is being most pointedly driven by the Europeans, who have produced a document entitled “Interpretation of Accreditation Requirements for Information Technology Test Laboratories for OSI Test Services.” It was decided by the workshop that, (a) the accreditation bodies are the responsible parties for bilateral and/or multilateral agreements, whether they are across the board or sector specific; and (b) a harmonized OSI—or more general FT—interpretation of accreditation requirements, is a necessary component of any such agreements. The development of that interpretation is the province of the OSI/IT communities, and the European document may already provide much of the input.

## 5. Certification

The discussions on certification were perhaps the most confusing and the least generative of a solution. Principal among the questions were: What is certification? and. Who wants it? Of course manufacturers worldwide would prefer first party certification, i.e., a “Manufacturer’s Declaration of Conformity,” in accordance with ISO Guide 22. Indeed, this may be adopted as a component of more independent product quaUty assurance schemes, however IT users seem to be unanimous in rejecting it in its raw, unequivocal “trust me” form. In Europe, the testing establishment is taking the lead in requirements setting, and hence the naturally favored solution is a third party certificate, from an independent testing organization, widely recognized by all buyers of OSI technology. Within the United States, the Corporation for Open Systems also favors the third party certificate approach, in the form of the COS Mark. NIST, in the United States, has established a testing program which has as a goal the development of a Register of Conformance Tested Products. In this case a register entry, based on review of an acceptable conformance test report, is raised, with no particular intention to generate a certificate. This difference in philosophy arises perhaps from the fact that NIST is not primarily acting as a third party certifier, but is representing and protecting the interests of the Federal Agencies as procurement authorities. The message which is conveyed in this case is that registered products have passed a basic set of qualification tests and can be considered for Federal procurement. There is no question of NIST, or the Federal Government, certifying or providing a warranty for products tested. Since the registers are public, other interested parties outside the government are free to use the information as they see fit.

## 6. Outlook

There are many problems to be solved before Worldwide Recognition of OSI Test Results can be achieved: adoption of common protocol specifications, development of complete test suites, harmonization of test system quality assurance methodologies, mutual recognition of accreditation schemes, and agreements on how all these components should fit together to provide assurance that an OSI product tested in one laboratory will be considered for procurement by any purchaser, anywhere in the world. An important outcome of this workshop is that consciousness has been raised over a wide international audience, of what the pitfalls are. The workshop also provided a stimulus to harmonizing test validation methodologies, and for the accreditation agencies to explore bilateral and multilateral agreements.

In conclusion, a report on the proceedings of the workshop will be presented to the ITRSG, and the workshop’s program committee has agreed to maintain contact in order to monitor progress on the above initiatives.

